# Association between gestational depression and weight management behaviors during pregnancy: A cross-sectional study in Eastern China

**DOI:** 10.3389/fpubh.2022.915786

**Published:** 2022-08-09

**Authors:** Shiqi Zhao, Xueqing Peng, Hua Zhou, Jinjin Ge, Meng Zhou, Anita Nyarkoa Walker, Hua You

**Affiliations:** ^1^School of Public Health, Nanjing Medical University, Nanjing, China; ^2^Changzhou Maternal and Child Health Care Hospital, Changzhou Medical Center, Nanjing Medical University, Changzhou, China; ^3^School of Nursing, Nanjing Medical University, Nanjing, China; ^4^Institute of Healthy Jiangsu Development, Nanjing Medical University, Nanjing, China

**Keywords:** weight management behaviors, association, gestational weight, depressive symptoms, pregnant women

## Abstract

An undesirable psychological state may deteriorate individual's weight management-related behaviors. This study aims to see if ineffective weight control measures were linked to depressive symptoms during pregnancy. We conducted a cross-sectional questionnaire survey of 784 pregnant women and collected information on sociodemographic factors, maternal characteristics, depression, and weight management activities throughout pregnancy (exercise management, dietary management, self-monitoring regulation, and management objectives). About 17.5% of pregnant women exhibited depressive symptoms. The mean score on dietary management was upper-middle, exercise management and self-monitoring regulation were medium, and management objectives were lower-middle. Multivariable linear regression analysis revealed that pregnant women with depressive symptoms had lower levels of exercise management (β = −1.585, *p* = 0.005), dietary management (adjusted β = −0.984, *p* = 0.002), and management objectives (adjusted β = −0.726, *p* = 0.009). However, there was no significant relationship between depressive symptoms and pregnant women's self-monitoring regulating behavior (*p* > 0.05). The findings indicated the inverse association between depressive symptoms and gestational weight management behaviors. These results offer important indications for pregnancy weight management professionals by highlighting the need for mental health interventions for pregnant women experiencing depressive symptoms.

## Introduction

Gestational weight gain (GWG) disorders are a prevalent health issue during pregnancy, particularly the rising trend of excess gestational weight gain (EGWG) (1), which has turned into a global public health issue and a significant economic burden. According to the 2009 American Institute of Medicine (IOM) Criteria, GWG above or below the guidelines for pregnant women reached 39.4 and 27.8%, respectively, according to a recent systematic review and meta-analysis (1). EGWG was found in 50.0% of pregnant women in 14 Chinese cities (*N* = 8,926) in retrospective research (2), which is greater than the global prevalence. GWG is a significant, modifiable risk factor for a variety of maternal and child health outcomes (3–5). As a result, it's critical to pay special attention to the issue of weight management during pregnancy.

An individual's weight-management behaviors can be influenced by an undesirable psychological state. Emotional eating (the tendency to eat in response to unpleasant emotions, frequently with a predilection for energy-dense and palatable meals) has been linked to depressive symptoms in studies (6). Dixon et al. discovered that moderate and severe depressive symptoms were connected with an unhealthy diet and reduced physical activity in obese diabetic patients (7). Jones et al. investigated the impact of mental health on obesity patients' attendance and engagement in behavioral weight management programs, finding that those with depressive symptoms at the start had lower participation rates throughout the program (8). The above research evidence indicated that the presence of depressive symptoms has an important impact on individual's weight management-related behaviors.

During pregnancy, depression is a frequent psychological illness. Pregnant women are more likely than the general population to develop depression symptoms as a result of specific physiological periods, changes in hormone levels, and environmental influences (9). Approximately 10% of pregnant women suffer from depression at some point throughout their pregnancy (10). Furthermore, as a result of the COVID-19 pandemic, pregnant women's depression symptoms increased dramatically (11). According to a meta-analysis, the prevalence of depression in pregnancy was 25.6% during the COVID-19 pandemic (12). We hypothesize that depressed symptoms induce pregnant women to be slack in weight control, resulting in disordered weight gain during pregnancy, based on current research on the relationship between depression and poor weight management behaviors in the general population. Following a study of the literature, we discovered that just a few research have validated this link. Do maternal weight management behaviors change as a result of depression symptoms during pregnancy? And what is the impact? This is something worth looking into.

Furthermore, according to the IOM (13), when it comes to weight management during pregnancy, there are usually two types of behaviors: a reasonable healthy diet and suitable physical activity. However, American researchers Kolodziejczyk et al. developed a weight management strategy scale as a tool for evaluating weight management behaviors in overweight/obese adults. The scale divides weight management behaviors into four categories: diet, physical activity, self-monitoring, and self-regulation (14). Then, based on this, Chinese researchers Yan et al. developed a pregnancy weight management strategy scale (PWMSS) for pregnant women and tested its efficiency (15). PWMSS analyzes more complete weight management behaviors during pregnancy than food and activity management, which will lead to a more in-depth study on weight management behaviors. This scale was used in a study on pregnant weight gain (16). As a result, portions of this scale were used in our research to look into weight management habits in pregnant women.

Specifically, we aimed to investigate the association between maternal depressive symptoms and pregnancy weight management behaviors adequacy and to provide evidence for targeted interventions for the management of GWG.

## Materials and methods

### Study setting and sampling

This cross-sectional survey was conducted in Changzhou City, Jiangsu Province, China. Changzhou is located in the southern part of more developed Jiangsu, is one of the central cities in the Yangtze River Delta region of China, covering an area of about 4,372.15 square kilometers. According to data released by the National Bureau of Statistics of China, in 2021, the permanent residents of Changzhou are 5,278,100 people, and the per capita GDP is 165,724 CNY (the national per capita GDP is 80,976 CNY).

We utilized a multi-stage stratified selection procedure to choose participants for this survey, which took place between August and December 2021. Three of Changzhou's five administrative regions were randomly selected in the first step, and two community health service facilities were randomly picked in each administrative region in the second stage. As a result, six community health service centers were chosen as survey sites; in the third stage, pregnant women who received routine prenatal care at the designated community health service centers during the investigation period and met the inclusion criteria were included. (1) These criteria included being within 14 weeks of pregnancy, (2) having a singleton pregnancy, (3) not having any medical comorbidities or a history of psychological illnesses, and (4) being able to complete the questionnaire independently.

Three uniformly trained investigators gathered all of the surveys. The investigators explained the study's goal and asked pregnant women to join while they waited for their prenatal examination in the outpatient clinic. The investigator distributed the questionnaire to the pregnant women after getting their informed consent. The participants voluntarily filled out the questionnaire. A questionnaire takes roughly 10–15 min to complete. The minimal sample size for this survey was 400, based on the sample size calculation formula of 10 times the number of questionnaire items plus 10% faulty questionnaires (17). A total of 814 pregnant women volunteered to take part in the study. After eliminating 30 surveys that were missing or poorly filled and did not pass the questionnaire quality check, the final analysis included 784 questionnaires with a 96.3% effective response rate. This investigation was approved by the Ethics Committee of Nanjing Medical University (No. (2020)63).

### Measurement

#### Sociodemographic and maternity characteristics

This part included age, educational level, working status, household monthly income per capita (CNY), pre-pregnancy weight, height, and parity.

#### The edinburgh postpartum depression scale

This is a useful tool for assessing maternal depression symptoms after childbirth. Although the EPDS was created to screen for postnatal depression, several researchers discovered that it might also detect antenatal depressive symptoms (18). It has been employed in several investigations of pregnant women suffering from prenatal depression (19, 20). Many languages have been translated and certified for EPDS. Wang et al. created a Chinese version of the EPDS based on Cox et al.'s scale (21), which was utilized in pregnancy research in China (22). As a result, we used the Chinese version of the EPDS to assess depression symptoms in pregnancy, and the scale's Cronbach's alpha was 0.809 in the study's population. The EPDS consists of 10 items. The items were accessed with “Never,” “Occasionally,” “Often,” and “Always,” which were rated as 0, 1, 2, or 3, corresponding to the intensity of the condition. The total score is calculated by summing the scores for each item, the maximum point is 30. The higher the score, the more severe the symptoms of depression are. Pregnant women gave their responses based on how they had been feeling for the previous 7 days.

#### Weight management scale

The PWMSS compiled by Yan et al. was used as a tool to measure the weight management behavior of pregnant women in the past 30 days, and it has good reliability and validity (15). For this study, we partially revised the scale by consulting experts. The revised scale has Cronbach's alpha of 0.855, Kaiser-Meyer-Olkin value of 0.894, and *p-*value of 0.001 for Bartlett's sphericity test. Exercise management, dietary management, self-monitoring regulation, and management objectives were the four dimensions of the revised scale. There are nine, four, four, and three elements in each dimension, and Cronbach's alpha are 0.812, 0.665, 0.665, and 0.877, respectively (Supplementary Table 1). Each item is rated on a 5-point Likert scale: 1 = never, 2 = seldom, 3 = occasionally, 4 = frequently, and 5 = always. As a result, the scoring range is 20–100 points, with individual score ranges of 9–45 points, 4–20 points, 4–20 points, and 3–15 points for each dimension, respectively. More pregnancy weight control behaviors are associated with higher scores.

### Study variables

#### Outcome variable

The outcome variables of this study were the four pregnancy weight management behaviors, including exercise management, dietary management, self-monitoring regulation, and management objectives. All of them were described with the average score of each subscale.

#### Explanatory variables

The explanatory variable in this study was the maternal depressive symptoms. Each version of the EPDS has cut-off values appropriate for their population. The cut-off value of 9.5 in identifying prevalence of probable depression in Chinese pregnant women has demonstrated satisfactory validity and reliability (22). Therefore we used this cut-off value to screen depressive symptoms during pregnancy.

#### Covariates

Sociodemographic and maternity characteristics were included as covariates (23–26) in the multivariable linear regression analysis. Age was categorized into two groups according to the age of high-risk pregnancy (18–34 years vs. ≥35 years); The highest educational level was categorized into three groups (middle school or lower, high school or vocational school, and associate degree or higher); Working status was categorized into two groups (employed vs. unemployed); Household monthly income per capita (CNY) was categorized into three groups (< 10,000, 10,000–20,000, and >20,000); Parity was categorized into two groups (primiparity vs. multiparity); Pre-pregnancy BMI was calculated based on the equation of BMI = weight (kg)/height (m)^2^. According to the cutoffs recommended in Weight Monitoring and Evaluation of Chinese Women during Pregnancy (27), it was categorized into four groups (underweight “ <18.5”, normal weight “18.5–23.9,” overweight “24.0–27.9,” and obese “≥28.0”).

### Statistical analysis

The frequencies and percentages were used to describe the sociodemographic and maternity characteristics of the participants. The mean and standard deviation were used to describe the EPDS score and the weight management behavior scores. *T*-test and analysis of variance (ANOVA) was used to analyze the differences of weight management behaviors during pregnancy in various demographic and maternity groups. Univariate Linear regression analyses were used to explore the correlation of depressive symptoms with four behavioral dimensions including exercise management (Model 1), dietary management (Model 2), self-monitoring regulation (Model 3), and management objectives (Model 4), respectively. Multivariable linear regression analysis was done by including depression status as the core independent variable and sociodemographic and maternity characteristics as covariates. All regression tests were performed using the Enter method, and statistical significance was set at *p* < 0.05. Data analysis was performed by using SPSS 21.0 for Windows (SPSS Inc., Chicago, IL).

## Results

### Basic characteristics, depression, and weight management measurements in participants

Overall, the mean (standard deviation) of participants age was 29.0 (3.7), with 7.0% of those aged 35 and above. In terms of educational level, 50.3% had a bachelor's degree or higher. The bulk of them was working, accounting for 89.4% of the total. Primiparity was found in 70.0% of the cases. The proportion of people with an average monthly household income (CNY) of < 10,000 yuan was 36.4%, 34.9% for 10,000–20,000 yuan, and 28.7% for more than 20,000 yuan. Pre-pregnancy BMI was within the normal range in 71.2% of pregnant women, 11.9% were underweight, 13.4% were overweight, and 3.6% were obese. The mean (standard deviation) of EPDS score was 6.6 (3.6), and 17.5% of the participants had EPDS scores higher than the cut-off value 9.5. The's mean (standard deviation) for WMS total score was 55.3 (12.7). And the mean (standard deviation) for the four WMS subscale scores were 21.7 (6.1), 12.8 (3.5), 10.3 (3.5), and 6.2 (3.0) separately (Table 1).

**Table 1:** Characteristics of participants (*N* = 784).

**Variables**	***N* (%)/mean (SD)**
**Age**	29 (3.7)
**Age group (years)**	
18–34	729 (93.0)
≥35	55 (7.0)
**Educational level**	
Middle school or lower	74 (9.4)
High or vocational school	98 (12.5)
Associate degree or higher	612 (78.1)
**Working status**	
Employed	701 (89.4)
Unemployed	83 (10.6)
**Household monthly income per capita (CNY)**	
<10,000	285 (36.4)
10,000–20,000	274 (34.9)
>20,000	225 (28.7)
**Parity**	
Primiparity	549 (70.0)
Multiparity	235 (30.0)
**Pre-pregnancy BMI**	
Underweight	93 (11.9)
Normal weight	558 (71.2)
Overweight	105 (13.4)
Obese	28 (3.6)
**EPDS score**	6.6 (3.6)
<9.5	647 (82.5)
≥9.5	137 (17.5)
**WMS score**	55.3 (12.7)
*Subscales*	
Exercise management	21.7 (6.1)
Dietary management	12.8 (3.5)
Self-monitoring regulation	10.3 (3.5)
Management objectives	6.2 (3.0)

CNY, Chinese Yuan (1 Chinese Yuan = 0.15 US Dollar).

### Pregnancy weight management behaviors of participants with different demographic characteristics

Table 2 shows that exercise management scores have significant statistical differences among different age groups (*t* = −4.715, *p* < 0.001), education levels (*F* = 6.946, *p* < 0.01), and working status (*t* = −3.801, *p* < 0.001); There are significant statistical differences in diet management scores among different educational levels (*F* = 33.506, *p* < 0.001), working status (*t* = −3.839, *p* < 0.001) and parity (*t* = 3.127, *p* < 0.01); The self-monitoring regulation scores have significant statistical differences among different educational levels (*F* = 16.691, *p* < 0.001) and working status (*t* = −3.607, *p* < 0.001).

**Table 2:** Pregnancy weight management behaviors of participants with different demographic characteristics.

**Characteristics**	**Exercise management**		**Dietary management**		**Self-monitoring regulation**		**Management objectives**	
	**Mean (SD)**	***t/F***	**Mean (SD)**	***t/F***	**Mean (SD)**	***t/F***	**Mean (SD)**	***t/F***
**Age**		−4.715***		−0.796		−1.444		−1.861
18–34	21.5 (5.9)		12.8 (3.5)		10.3 (3.5)		6.1 (2.9)	
≥35	25.4 (6.9)		13.2 (3.6)		11.0 (3.2)		7.0 (3.4)	
**Educational level**		6.946**		33.506***		16.691***		1.342
Middle school or lower	19.8 (6.3)		10.8 (3.5)		9.0 (3.4)		5.7 (2.8)	
High or vocational school	20.7 (5.4)		11.0 (3.4)		9.0 (3.5)		6.0 (2.8)	
Associate degree or higher	22.2 (6.1)		13.3 (3.4)		10.7 (3.4)		6.3 (3.0)	
**Working status**		−3.801***		−3.839***		−3.607***		−0.754
Unemployed	19.4 (5.5)		11.4 (3.6)		9.0 (3.2)		6.0 (3.2)	
Employed	22.0 (6.1)		13.0 (3.5)		10.5 (3.5)		6.2 (2.9	
**Household monthly**		0.820		2.526		2.910		0.944
**income per capita**							
<10,000	21.5 (5.7)		12.5 (3.6)		9.9 (3.5)		6.0 (2.9)	
10,000–20,000	21.7 (6.2)		13.2 (3.5)		10.4 (3.5)		6.2 (3.1)	
>20,000	22.2 (6.4)		12.8 (3.5)		10.7 (3.4)		6.4 (2.9)	
**Pre-pregnancy BMI**		1.637		1.028		1.195		0.567
Underweight	20.9 (6.2)		12.3 (3.5)		10.0 (4.0)		6.4 (3.1)	
Normal weight	22.0 (6.0)		12.9 (3.6)		10.4 (3.5)		6.2 (3.0)	
Overweight	21.5 (6.4)		12.6 (3.4)		10.2 (3.0)		6.2 (2.7)	
Obesity	20.2 (5.0)		12.9 (3.3)		9.4 (3.2)		5.5 (2.6)	
**Parity**		−1.754		3.127**		1.263		−0.627
Primiparity	21.5 (6.1)		13.1 (3.5)		10.4 (3.5)		6.1 (2.9)	
Multiparity	22.3 (6.1)		12.2 (3.6)		10.1 (3.4)		6.3 (3.0)	

**p < 0.01.

***p < 0.001.

### Crude association between EPDS score and weight management behaviors

In Table 3, the results of the univariate regression model showed that higher EPDS score (≥9.5) was significantly associated with lower scores for exercise management (β = −1.755, *p* = 0.002), diet management (β = −1.246, *p* < 0.001), and management objectives (β = −0.753, *p* = 0.007). EPDS score had no significant correlation with scores of self-monitoring regulation (*p* > 0.05; Table 3).

**Table 3:** Linear regression analysis for EPDS and WMS scores.

**EPDS score**	**Model 1**	**Model 2**	**Model 3**	**Model 4**
	**β (95% CI)**	**β (95% CI)**	**β (95% CI)**	**β (95% CI)**
**<9.5**	1	1	1	1
≥9.5	−1.755 (−2.869, −0.641)**	−1.246 (−1.892, −0.599)***	−0.631 (−1.274, 0.013)	−0.753 (−1.298, −0.208)**

**p < 0.01.

***p < 0.001.

### Multivariable linear regression analyses for EPDS scores and weight management behaviors

After adjusting for background variables, higher EPDS score (≥9.5) was significantly associated with lower scores for exercise management (β = −1.585, *p* = 0.005), diet management (β = −0.984, *p* = 0.002), and management objectives (β = −0.726, *p* = 0.009). EPDS score had no significant correlation with self-monitoring regulation (*p* > 0.05; Table 4).

**Table 4:** Multivariable linear regression analysis for EPDS and WMS scores.

**Characteristics**	**Model 1**	**Model 2**	**Model 3**	**Model 4**
	**β (95% CI)**	**β (95% CI)**	**β (95% CI)**	**β (95% CI)**
**EPDS score (Ref:** ** <9.5)**	−1.585 (−2.678, −0.493)**	−0.984 (−1.609, −0.359)**	−0.458 (−1.090, 0.175)	−0.726 (−1.274, −0.178)**
**Age (Ref: 18–34)**	3.689 (1.994, 5.384)***	0.842 (−0.128, 1.811)	0.874 (−0.107, 1.856)	0.855 (0.004, 1.706)*
**Educational level (Ref: middle school or lower)**				
High or vocational school	0.732 (−1.068, 2.531)	0.189 (−0.840, 1.218)	−0.109 (−1.150, 0.933)	0.158 (−0.745, 1061)
Associate degree or higher	2.314 (0.781, 3.846)**	2.193 (1.317, 3.069)***	1.450 (0.563, 2.338)***	0.534 (−0.235, 1.303)
**Working status (Ref: employed)**	1.649 (0.241, 3.056)*	0.671 (−0.133, 1.476)	−0.792 (−0.023, 1.607)	0.031 (−0.675, 0.738)
**Household monthly income per capita (Ref:** ** <10,000)**				
10,000–20,000	−0.038 (−1.017, 0.941)	0.540 (−0.020, 1.100)	0.388 (−0.179, 0.955)	0.150 (−0.341, 0.642)
>20,000	0.366 (−0.671, 1.402)	0.224 (−0.368, 0.817)	0.668 (0.068, 1.268)*	0.286 (−0.234, 0.806)
**Pre-pregnancy BMI (Ref: normal weight)**				
Underweight	−0.602 (−1.911, 0.706)	−0.508 (−1.257, 0.240)	−0.306 (−1.063, 0.452)	0.285 (−0.372, 0.941)
Overweight	−0.410 (−1.648, 0.827)	−0.054 (−0.762, 0.653)	−0.072 (−0.789, 0.644)	0.065 (−0.557, 0.686)
Obesity	−1.493 (−3.741, 0.755)	0.183 (−1.102, 1.469)	−0.782 (−2.083, 0.520)	−0.613 (−1.741, 0.515)
**Parity (Ref: primiparity)**	0.802 (−0.194, 1.797)	−0.403 (−0.972, 0.166)	−0.103 (−0.680, 0.473)	0.160 (−0.339, 0.660)

*p < 0.05.

**p < 0.01.

***p < 0.001.

## Discussion

In this survey sample, 17.5% of pregnant women was initially screened for depressive symptoms, which was lower than the findings of a nationwide maternal mental health cohort study in China (26.2%) (28). However, this is a fairly high percentage when compared to the prevalence of depression in China's general population (2.1%) (29). This demonstrates that pregnant women are at a high risk of developing depression. Meanwhile, this study found that pregnant women with depressive symptoms had poorer gestational weight management behavior scores on exercise management, dietary management, and management objectives than those without depressive symptoms. This negative correlation between depression and weight management strategies was consistent with prior findings in other populations, such as the general public (30), diabetics (7), obese people (8), etc. Thus, the problem of weight gain in pregnant women appears to be not only weight management *per se*, but also depression-related psychological and behavior disorders.

Pregnant women with depressive symptoms self-reported less physical activity. A review of depression and sedentary behavior found that patients with depression at baseline were more likely to develop a sedentary lifestyle in the future. They were also less likely to follow doctor's recommendations for physical activity (31, 32). Possible reasons are depressive symptoms damage a patient's mental health and physical performance, making them less willingness and uninterested in external activities (33). As a result of their negative emotions, these pregnant women may be hesitant to engage in various physical activities. On the other hand, people with depression are sensitive to pressure from their family and friends to be physically active (32). Adopting strategies to reduce sedentary behavior and getting more social support may be feasible paths to reduce depressive symptoms and increase physical activity.

Similarly, depressive emotions was significantly associated with pregnant women's eating habits. Lack of appetite and weight loss are two of the most common symptoms of depression (34). However, there are certain cases where depression can result in a large increase in hunger in some people (35). Regardless of the consequences, this is incompatible with their logical dietary management. According to Russo et al.'s research, depression is a risk factor for the occurrence of eating disorders in female teenagers seeking weight control (36). Another cohort-based cross-sectional secondary analysis found that pregnant women with prenatal depression consumed more calories, ate fewer vegetables, legumes, and fruits, and were more likely to have poor diet quality (37). Both these previous pieces of evidence and the results of this study suggest a potential impact of depression on pregnant women regarding diet control behaviors.

In addition, regarding management objectives and self-monitoring regulation behaviors, which are different from the direct ways of controlling weight (exercise and diet), they reflect the self-management and self-regulation ability of pregnant women for controlling weight. Bandura believed that humans have the willpower to “endure transient negative outcomes in exchange for long-term positive outcomes” (38). This willpower can be embodied in the fact that people set and complete phased goals, or regularly examine their behavior with goals and plans (39). According to a Canadian study, the most common barrier to making health-related behavioral adjustments in adults with depression was a lack of willpower (40). The self-management of weight management during pregnancy is a long-term and ongoing procedure, such high health demands reduce the self-efficacy of pregnant women to engage in more weight control activities, as well as the willpower to self- management. If a pregnant woman suffers from depression at the same time, it will increase health demands (41), which will consume more willpower, resulting in poor results in their management objectives behavior. However, we found no effect of depressed symptoms on self-monitoring regulation, which has to be investigated further.

According to the results of this study, being depression status is associated with a variety of weight management behaviors in pregnant women. Maternal health providers must consider the mental health of pregnant women. In weight intervention of pregnant women with depression, the strategies taken into account should involve alleviating the depressive symptoms of pregnant women and increasing their social support.

There were several limitations for this study as well. First, respondents' self-reports of completing the questionnaire were used to collect data for this study, which could lead to some information bias and thus alter the accuracy of the results. Second, because this was a cross-sectional study, it was unable to demonstrate a causal link, and further research is needed to confirm the findings. Furthermore, this study was conducted in a city in China and only recruited pregnant women within first 14 weeks of pregnancy, which may restrict the finding's generalizability. As a result, more research on the impact of pregnant women's depression state on weight management behavior is still needed. Despite these evident limitations, this study contributes to our understanding of gestational weight management.

## Conclusion

This study discovered a link between pregnant women's depressed psychological states and a variety of gestational weight management behaviors (including exercise management, dietary management, and management objectives), laying the groundwork for future interventions aimed at improving gestational weight management. Given the high prevalence of pregnant depressive symptoms and the importance of healthy GWG, maternal healthcare providers should not only focus on mechanically improving weight-control-related behaviors in pregnant women but also on their mental health status to carry out adaptive interventions, particularly for those pregnant women who have depressive symptoms.

## Data availability statement

The raw data supporting the conclusions of this article will be made available by the authors, without undue reservation.

## Ethics statement

The studies involving human participants were reviewed and approved by the Ethics Committee of Nanjing Medical University (No. (2020)63). The patients/participants provided their written informed consent to participate in this study.

## Author contributions

HY designed the study and critically reviewed, commented, and revised important intellectual content. SZ and XP performed the final statistical analyses and interpretation of data, and drafted the article. SZ, HZ, JG, and MZ collected the data. AW revised the draft grammatical sentences. All authors contributed to the article and approved the submitted version.

## Funding

This work was supported by National Natural Science Foundation of China (No. 72074122), Jiangsu Provincial Maternal and Child Health Research Project (No. F202028), Cultivation Project of Decision-making Consultation, Institute of Healthy Jiangsu Development, Nanjing Medical University.

## Conflict of interest

The authors declare that the research was conducted in the absence of any commercial or financial relationships that could be construed as a potential conflict of interest.

## Publisher's note

All claims expressed in this article are solely those of the authors and do not necessarily represent those of their affiliated organizations, or those of the publisher, the editors and the reviewers. Any product that may be evaluated in this article, or claim that may be made by its manufacturer, is not guaranteed or endorsed by the publisher.

## References

[1] Martínez-Hortelano JA Cavero-Redondo I Álvarez-Bueno C Garrido-Miguel M Soriano-Cano A Martínez-Vizcaíno V. Monitoring gestational weight gain and prepregnancy BMI using the 2009 IOM guidelines in the global population: a systematic review and meta-analysis. BMC Pregnancy Childbirth. (2020) 20:649. 10.1186/s12884-020-03335-7 33109112 PMC7590483

[2] Wang X Zhang X Zhou M Juan J Wang X. Association of prepregnancy body mass index, rate of gestational weight gain with pregnancy outcomes in Chinese urban women. Nutr Metab. (2019) 16:54. 10.1186/s12986-019-0386-z 31452666 PMC6700840

[3] Voerman E Santos S Inskip H Amiano P Barros H Charles M-A. Association of gestational weight gain with adverse maternal and infant outcomes. JAMA. (2019) 321:1702–15. 10.1001/jama.2019.3820 31063572 PMC6506886

[4] Catalano PM Shankar K. Obesity and pregnancy: mechanisms of short term and long term adverse consequences for mother and child. The BMJ. (2017) 356:j1. 10.1136/bmj.j1 28179267 PMC6888512

[5] Goldstein RF Abell SK Ranasinha S Misso M Boyle JA Black MH. Association of gestational weight gain with maternal and infant outcomes. JAMA. (2017) 317:2207–25. 10.1001/jama.2017.3635 28586887 PMC5815056

[6] Konttinen H Männistö S Sarlio-Lähteenkorva S Silventoinen K Haukkala A. Emotional eating, depressive symptoms and self-reported food consumption. A population-based study. Appetite. (2010) 54:473–9. 10.1016/j.appet.2010.01.014 20138944

[7] Dixon JB Browne JL Mosely KG Rice TL Jones KM Pouwer F. Severe obesity and diabetes self-care attitudes, behaviours and burden: implications for weight management from a matched case-controlled study. Results from Diabetes MILES—Australia. Diabet Med. (2014) 31:232–40. 10.1111/dme.12306 23952552

[8] Jones RA Mueller J Sharp SJ Vincent A Duschinsky R Griffin SJ. The impact of participant mental health on attendance and engagement in a trial of behavioural weight management programmes: secondary analysis of the WRAP randomised controlled trial. Int J Behav Nutr Phys Act. (2021) 18:146. 10.1186/s12966-021-01216-6 34743721 PMC8574009

[9] Howard LM Molyneaux E Dennis C-L Rochat T Stein A Milgrom J. Non-psychotic mental disorders in the perinatal period. Lancet. (2014) 384:1775–88. 10.1016/S0140-6736(14)61276-9 25455248

[10] Vigod SN Wilson CA Howard LM. Depression in pregnancy. BMJ. (2016) 352:i1547. 10.1136/bmj.i1547 27013603

[11] Lebel C MacKinnon A Bagshawe M Tomfohr-Madsen L Giesbrecht G. Elevated depression and anxiety symptoms among pregnant individuals during the COVID-19 pandemic. J Affect Disord. (2020) 277:5–13. 10.1016/j.jad.2020.07.126 32777604 PMC7395614

[12] Tomfohr-Madsen LM Racine N Giesbrecht GF Lebel C Madigan S. Depression and anxiety in pregnancy during COVID-19: a rapid review and meta-analysis. Psychiatry Res. (2021) 300:113912. 10.1016/j.psychres.2021.113912 33836471 PMC9755112

[13] Rasmussen KM Yaktine AL Institute Institute of Medicine (US) National Research Council (US) Committee to Reexamine IOM Pregnancy Weight Guidelines. Weight Gain During Pregnancy: Reexamining the Guidelines. Washington, DC: National Academies Press (2009). Available online at: http://www.ncbi.nlm.nih.gov/books/NBK32813/ (accessed March 23, 2022).

[14] Kolodziejczyk JK Norman GJ Roesch SC Rock CL Arredondo EM Madanat H. Exploratory and confirmatory factor analyses and demographic correlate models of the strategies for weight management measure for overweight or obese adults. Am J Health Promot. (2015) 29:e147–57. 10.4278/ajhp.130731-QUAN-391 24670075

[15] Yan J Zhu X Kong X Wang T. Development, reliability and validity test of pregnancy weight management strategy scale. Int Med Health Guid News. (2017) 23:2632–5. 10.3760/cma.j.issn.1007-1245.2017.16.04

[16] Zixin XI Fanghua GO Fang TA Lichun WA Jiaxing LI. Correlation analysis of pregnant women's self-management, strategy for weight management and weight control during pregnancy. Nurs Integr Tradit Chin West Med. (2020) 6:31–6. 10.11997/nitcwm.202002006

[17] Chin WW. “*The Partial Least Squares Approach for Structural Equation Modeling,” Modern Methods for Business Research*. Methodology for Business and Management. Mahwah, NJ: Lawrence Erlbaum Associates Publishers (1998). p. 295–336.

[18] Murray D Cox JL. Screening for depression during pregnancy with the edinburgh depression scale (EDDS). J Reprod Infant Psychol. (1990) 8:99–107. 10.1080/02646839008403615

[19] Bunevicius A Kusminskas L Pop VJ Pedersen CA Bunevicius R. Screening for antenatal depression with the Edinburgh Depression Scale. J Psychosom Obstet Gynaecol. (2009) 30:238–43. 10.3109/01674820903230708 19845492

[20] Evans J Heron J Francomb H Oke S Golding J. Cohort study of depressed mood during pregnancy and after childbirth. BMJ. (2001) 323:257–60. 10.1136/bmj.323.7307.257 11485953 PMC35345

[21] Cox JL Holden JM Sagovsky R. Detection of postnatal depression: development of the 10-item edinburgh postnatal depression scale. Br J Psychiatry. (1987) 150:782–6. 10.1192/bjp.150.6.782 3651732

[22] Wang Y Guo X Lau Y Chan KS Yin L Chen J. Psychometric evaluation of the Mainland Chinese version of the Edinburgh Postnatal Depression Scale. Int J Nurs Stud. (2009) 46:813–23. 10.1016/j.ijnurstu.2009.01.010 19217107

[23] Zhan YL Shi YJ Chen Y Feng YH Wu S Wang Y. Influence of reproduction history on depression during pregnancy: a prospective cohort study. Chin J Dis Control Prev. (2020) 24:324–9. 10.16462/j.cnki.zhjbkz.2020.03.015

[24] Hill B Skouteris H McCabe M Milgrom J Kent B Herring SJ. conceptual model of psychosocial risk and protective factors for excessive gestational weight gain. Midwifery. (2013) 29:110–4. 10.1016/j.midw.2011.12.001 23159235

[25] American Institute Of Medicine. Influence of Pregnancy Weight on Maternal Child Health: A Workshop Report. (2007). Available online at: https://nap.nationalacademies.org/read/11817/chapter/8 (accessed March 14, 2022).

[26] Puente CP Monge FJC Abellán IC Morales DM. Effects of personality on psychiatric and somatic symptoms in pregnant women: the role of pregnancy worries. Psychol Women Q. (2011) 35:293–302. 10.1177/0361684310384105

[27] Chinese Nutrition Society. Announcement of Chinese Nutrition Society on Publishing Two Group Standards. (2021). Available online at: https://www.cnsoc.org/otherNotice/392100200.html (accessed March 23, 2022).

[28] Yang YH Huang X Sun MY Yang L Zheng RM. Analysis on depression state outcomes and influencing factors of persistent depression in pregnant and perinatal women in China. Chin J Epidemiol. (2022) 43:58–64. 35130653 10.3760/cma.j.cn112338-20210628-00502

[29] China, Releases. National Health Commission: The Prevalence of Depression in my Country is 2.1%, and the Prevalence of Anxiety Disorders is 4.98%. Available online at: http://news.china.com.cn/txt/2020-12/23/content_77043277.htm (accessed March 25, 2022).

[30] Difrancesco S Lamers F Riese H Merikangas KR Beekman ATF van Hemert AM. Sleep, circadian rhythm, and physical activity patterns in depressive and anxiety disorders: a 2-week ambulatory assessment study. Depress Anxiety. (2019) 36:975–86. 10.1002/da.22949 31348850 PMC6790673

[31] Roshanaei-Moghaddam B Katon WJ Russo J. The longitudinal effects of depression on physical activity. Gen Hosp Psychiatry. (2009) 31:306–15. 10.1016/j.genhosppsych.2009.04.002 19555789

[32] Schuch F Vancampfort D Firth J Rosenbaum S Ward P Reichert T. Physical activity and sedentary behavior in people with major depressive disorder: a systematic review and meta-analysis. J Affect Disord. (2017) 210:139–50. 10.1016/j.jad.2016.10.050 28033521

[33] Lim G. Perinatal depression. Curr Opin Anesthesiol. (2021) 34:233–7. 10.1097/ACO.0000000000000998 33935170 PMC8098643

[34] Malhi GS Mann JJ. Depression. Lancet. (2018) 392:2299–312. 10.1016/S0140-6736(18)31948-2 30396512

[35] Levitan RD Davis C Kaplan AS Arenovich T Phillips DIW Ravindran AV. Obesity comorbidity in unipolar major depressive disorder: refining the core phenotype. J Clin Psychiatry. (2012) 73:12987. 10.4088/JCP.11m07394 22687640

[36] Russo JV Brennan L Walkley J Fraser SF Greenway K. Psychosocial predictors of eating disorder risk in overweight and obese treatment-seeking adolescents. Behav Change. (2011) 28:111–27. 10.1375/bech.28.3.111

[37] Avalos LA Caan B Nance N Zhu Y Hyde RJ Li D-K. Prenatal depression and diet quality during pregnancy. J Acad Nutr Diet. (2020) 120:972–84. 10.1016/j.jand.2019.12.011 32063456 PMC8006531

[38] Bandura A. Social learning theory of aggression. J Commun. (1978) 28:12–29. 10.1111/j.1460-2466.1978.tb01621.x 690254

[39] Fu Hua. Health Education. 3rd edition. People's Medical Publishing House (2017). p. 420. Available online at: https://book.douban.com/subject/35668334/ (accessed March 25, 2022).

[40] Clayborne ZM Colman I. Associations between depression and health behaviour change: findings from 8 cycles of the canadian community health survey. Can J Psychiatry Rev Can Psychiatr. (2019) 64:30–8. 10.1177/0706743718772523 29734818 PMC6364138

[41] Di Maio S Keller J Job V Felsenberg D Ertel W Schwarzer R. Health demands moderate the link between willpower beliefs and physical activity in patients with knee osteoarthritis. Int J Behav Med. (2020) 27:406–14. 10.1007/s12529-020-09865-w 32162213 PMC7359122

