# Supplementary material for: Association between gestational depression and weight management behaviors during pregnancy: A cross-sectional study in Eastern China
Source: Front Public Health. 2022 Aug 9;10:915786. doi: 10.3389/fpubh.2022.915786 (PMC9395704; doi:10.3389/fpubh.2022.915786)
Supplement: Supplementary file 1 [file Table_1.docx]

**Supplementary Table 1. Weight management scale**

| **Here is the weight management strategy scale for pregnancy. Please answer the following questions according to the situation in the past month** **(only fill in the corresponding number):**  **①Never ②Rarely ③Sometimes ④Often ⑤Always** | | |
| --- | --- | --- |
|  | Even without a gym or sports equipment, I still exercise |  |
|  | I exercise for 30 minutes or more every day |  |
|  | I monitor the number of steps daily with a pedometer or stepping software |  |
|  | I keep exercising without the encouragement of my family and friends |  |
|  | I don't reduce exercise as my belly gradually gets bigger after pregnancy |  |
|  | When sports encounter difficulties, I can find a way to overcome them |  |
|  | I insist on exercise even when I am tired or in a bad mood |  |
|  | I continue to work during pregnancy and do light housework normally |  |
|  | I found the suitable type of exercise for me during pregnancy |  |
|  | I don’t store high-calorie foods or favorite foods at home |  |
|  | I reduce the intake of fried, high fat food and sugary drinks |  |
|  | I will not eat too much at dinner |  |
|  | I will not watch TV while eating |  |
|  | I take measures to control my weight when my weekly weight gained too much |  |
|  | I measure my weight once a week and record it |  |
|  | I record my daily diet and exercise |  |
|  | On a scale of 1 to 5, 1 is having no control over what you eat, and 5 is having complete control over. What level are you in during pregnancy |  |
|  | I make my weekly weight gain plan and check whether my gestational weight gain is up to standard |  |
|  | I make my own diet plan and check if I eat as planned |  |
|  | I make my own sports plan and check if I follow it |  |
